# Supplementary material for: Are carbon emissions trading and green financial instruments synergistic? -Comprehensive quantitative research based on content analysis
Source: PLoS One. 2024 Mar 7;19(3):e0298601. doi: 10.1371/journal.pone.0298601 (PMC10919601; doi:10.1371/journal.pone.0298601)
Supplement: S1 Data — (ZIP) [file pone.0298601.s001.zip › Data/地级市Z_F工作报告.zip!/╕ú╜¿/╚¬╓▌╩╨/└┤╫╘╣1⁄2╓┌║┼í╛┬φ┐╦╩2╛▌═°í┐_2008.docx]

各位代表：

现在，我代表泉州市人民政府向大会作工作报告，请予审议，并请政协委员和其他列席人员提出意见。

一、2007年政府工作回顾

2007年是本届政府的第一年，也是实施“十一五”规划的重要一年。我市各级政府认真落实科学发展观，服务海峡西岸经济区建设大局，坚持市委“好中求快、支撑带动”工作主线，扎实推进“七个围绕”重点工作，顺利实现了市十四届人大一次会议确定的经济社会发展目标。全年实现生产总值2288.60亿元，增长16.0%；财政总收入225.06亿元，增长22.8%，其中，一般预算收入114.61亿元，增长23.2%。晋江、惠安、石狮、南安、安溪5县（市）进入第七届全国县域经济基本竞争力百强县（市），所有县（市）均保持全省经济实力十强或经济发展十佳。荣获中国优秀创新型城市、全国科技进步先进城市“六连冠”、全国双拥模范城“六连冠”、市民最满意城市、感动世界的中国品牌城市等称号。一年来的主要工作和成效是：

新农村建设“五大工程”深入实施，“三农”工作取得新成效。认真落实市委、市政府《关于加快推进社会主义新农村建设的实施意见》和《泉州市社会主义新农村建设规划纲要（2006—2010年）》，推进省市县三级试点村和帮扶村建设，投入新农村建设资金15.76亿元。大力发展现代农业，扎实推进“1333”工程，新增省级农业产业化龙头企业3家，新增无公害农产品、绿色食品、有机食品企业48家、产品65个，成功举办泉州龙眼（北京）展览推介会。加强粮食储备安全和粮食市场体系建设，兑付种粮农民综合直补资金3700万元。有效防抗“6·19”特大暴雨和“帕布”、“圣帕”等台风。做好牲畜口蹄疫、高致病性禽流感等重大动物疫病防控工作。完成“六千”水利工程、山美灌区节水改造、农村沼气工程等年度建设任务，建成海上渔业安全管理信息系统。实现移动电话“村村通”。完成集体林权制度改革发（换）证工作。出台《海峡两岸（泉州）农业合作试验区发展规划》，成功举办首届海峡两岸茶业博览会。实施“阳光工程”，转移农村劳动力6.7万人。实施“造福工程”，搬迁5650人。完成124个省、市确定的村级组织活动场所建设任务。

自主创新能力不断增强，产业结构优化升级步伐加快。认真实施市委、市政府《关于增强自主创新能力的决定》，新增国家级企业技术中心1家和省级企业技术中心13家、行业技术开发中心13家、高新技术企业46家，“6·18”项目成果交易会成功对接项目598个。传统产业改造提升步伐加快，新增上市企业7家；规模以上工业产值占工业总产值比重达80.9%，提高4.3个百分点；新增中国驰名商标（国家工商总局认定）6件、中国名牌产品7件、国家出口免验产品3件。新兴产业发展迅速，石油化工、修船造船、汽车制造业产值分别增长11.5%、591.9%、52.9%；鲤城、南安、惠安、晋江等县（市、区）光电信息产业基地建设步伐加快；福建联合石化公司挂牌运营，完成投资106亿元；泉州船舶工业公司2艘万吨级油轮顺利下水；一汽新华旭生产各类专用汽车1200辆，五洲龙汽车制造、锐驰汽车电子项目开工建设；海裕生物制药等项目投产。全市新增产值超亿元企业138家。服务业稳步发展，第三产业增加值增长14.8%；实现旅游总收入172.40亿元，增长18.2%；金融机构本外币存款余额1873.77亿元，贷款余额1417.49亿元，分别比年初增长11.3%和24.0%。

重点项目建设顺利推进，城市发展空间进一步拓展。126个市级在建重点项目完成投资177亿元。金鸡拦河闸重建、洛秀组团东西主干道等19个项目建成投产；泉三高速公路泉州段路基工程、泉州市海峡体育中心“一场一馆”等31个项目主体完工；福厦铁路泉州段、泉州液化天然气配套管网、外走马埭围垦工程等项目进展顺利，中化重油深加工、泉州船厂造船等33个项目开工建设；电网建设完成投资15亿元，新塘等15个220千伏输变电工程项目投入运行。“一二三四”城建项目扎实推进，《泉州市城市总体规划》（修编）通过审查，《泉州市古城保护整治规划》获批；泉州大桥南立交桥和朋山岭第二隧道及连接线建成通车，中心市区内环路主体工程基本完工；城东、东海、桥南三大片区建设加快推进，中心市区建成区面积扩大6平方公里，全市城镇化率达53%。认真执行市十四届人大一次会议议案决议，大力推进城市道路交通综合整治，试行错时上下班制度，完成部分路段单行道、人行天桥建设和70条街巷路面改造，加强交通疏导和管理，建成江南等一批公交站场，投入4757万元新购公交车155部，组建第二公交公司，开通特许经营线路，中心市区道路交通拥堵现象有所缓解。开展城市管理相对集中行政处罚权工作，“两违”现象得到有效遏制。港城联动步伐加快，重点港区控制性详规和部分港后区规划编制基本完成，实现港口投资11.7亿元，完成港口货物吞吐量6215.32万吨，集装箱吞吐量突破100万标箱。

开放型经济向更高层次发展，对外开放和对台经贸合作力度进一步加大。全市实际利用外资（验资口径）10.85亿美元，增长17.9%。新批投资（含增资）总额超千万美元项目124个。出口加工区建成进入招商选资阶段，申报台商投资区工作扎实推进。打造台资聚集区取得突破，和谐光电、长照科技等一批重大台资项目相继落地。成功举办泉台产业合作投资洽谈会，泉澎货运直航实现常态化，泉澎客运首航成功，泉金客运航线航班增加，客源增长183%。加强外经贸支撑服务体系建设，全年实现出口商品总值（海关口径）49.81亿美元，增长23.4%。继续深化泉港合作八大平台建设和泉澳四项合作。提高山海协作、城市联盟和对口支援工作水平。

环境整治和保护力度加大，资源节约型和环境友好型社会建设稳步推进。大力实施市委、市政府《关于进一步加强环境保护工作的决定》，认真执行市十三届人大七次会议议案决议。近海水域污染整治投入资金10.8亿元，完成84个年度整治项目。“两江”上游水资源保护投入政府专项资金2800万元，完成整治项目38个。各县（市、区）城市生活污水处理厂建成投入运行或试运行，中心市区污水处理率和垃圾无害化处理率分别达到77%和98%左右，各县（市、区）污水、垃圾处理全部实行产业化运作。完成循环经济示范项目29个，亿元工业产值电耗比上年减少23.94万千瓦时。去年初确定的节能减排目标预计可以完成。严格保护耕地，大力推进土地资源的集约节约利用，完成土地整理9200亩。全面推进经营性用地和工业用地“招拍挂”，开展第二次全国土地调查和新一轮土地利用总体规划修编。抓好潜在地质安全隐患点及地质灾害点调查复核工作。市级海洋功能区划获省政府批准。

社会各项事业协调发展，文化强市建设迈出新步伐。坚持教育优先发展。全市落实“两免一补”经费1.93亿元，惠及86.1万名学生，外来务工人员子女平等接受义务教育工作走在全省前列。基础教育课程改革全面推进，鲤城被确定为全国社区教育实验区，新增惠安、德化县实现省“双高普九”，中小学危房改造新建校舍10.41万平方米。高等教育继续发展，泉州慈山财经学校并入泉州经贸学院。积极开展招才引智工作，组织用人单位赴合肥、兰州、南京、大连等地招聘人才，颁发首届“泉州市杰出人才奖”，新增博士后工作站2家。大力发展文化事业。5个县（区）获得省级文化先进县（区）称号。梨园古典剧院、南音艺苑建设基本完成，东西塔保护、承天寺景观修复工程和清净寺新礼拜堂建设顺利实施。国家级闽南文化生态保护实验区、中国民族民间文化保护试点城市工作扎实开展。文艺创作持续繁荣，3件作品获全国群星奖。文物和非物质文化遗产保护工作获得13项国家级表彰。泉台文化双向交流保持活跃，闽南语电视频道开播，中国闽台缘博物馆接待国内外游客55.68万人次，其中台胞4.86万人次。切实加强卫生事业。参加新型农村合作医疗人数423.48万人，占全市农村人口的82%。市第一医院新院建设、市人民医院新院复建和22所重点乡（镇）卫生院综合配套建设加快推进。积极开展新一轮文明城市创建活动。扎实推进第六届全国农运会筹备工作。成功举办第八届市运会。全市人口出生率10.42‰，政策符合率94.61%，继续保持低生育水平。哲学社会科学、新闻出版、青少年事务、妇女儿童、老龄、残疾人、档案、审计、统计、修志、民族宗教、外事侨务、气象、防震减灾、国防动员、双拥、支前、民兵、预备役、海防、人防、打私、口岸等工作取得新成绩。

发展成果进一步惠及人民群众，社会继续保持和谐安定。全市城镇居民人均可支配收入和农民人均纯收入达18097元和7244元，分别增长13.3%和9.7%。城乡就业再就业工作得到加强，城镇新增就业15.8万人，城镇登记失业率1.2%。社保覆盖面进一步扩大，11.24万人享受城乡低保，30万外来务工人员、农民工参加职工医疗互助。提高最低工资标准，90%企业实行劳动合同制。调整企业养老金标准，6.3万名企业退休人员人均每月增加90.69元。努力构建新型社会救助体系，全市累计认捐慈善基金10亿元。基本完成中侨公司、燃料公司等国有企业改制职工安置工作，市属国有、集体困难企业职工全部参加基本医疗保险。住房保障全面启动，中心市区安置房、限价房、廉租房建设步伐加快。抓好产品质量和食品安全专项整治行动。建设覆盖城乡的“12315”消费维权网络。深化“平安泉州”创建活动，建立流动人口服务管理新机制，推进社会治安综合治理，深入开展禁毒人民战争和“打黑除恶”、“反盗抢”、“扫黄打非”、打击虚假信息诈骗等专项斗争，社会继续保持安定稳定，实现“ 一升 一降”目标。完善信访长效工作机制，妥善化解基层矛盾纠纷。推进“五五”普法，开展人民调解“十百千万”示范活动。推进安全生产11个方面专项整治，事故起数、死亡人数分别下降	9.12%和6.26%。

行政审批制度改革持续深化，政府机关效能进一步提升。市行政服务中心进一步规范运作，42个部门和“六大中心”入驻中心办公，546项审批服务项目进入中心审批，20个主要部门专设审批科成建制进入中心运作，推行全程式网上审批，实行并联审批，项目审批时限缩短近五分之四。切实加强公共应急管理工作，进一步完善应急预案、应急体制机制建设。扎实推进政务公开、政府绩效评估和规范行政处罚自由裁量权试点工作。持续深化反腐倡廉工作，加大案件查处力度，处理违法违纪政府机关工作人员96人，挽回经济损失273.04万元。支持和保障人大代表、政协委员依法开展工作，办理人大代表建议 366件、政协委员提案439件，办复率均达100%，满意率分别达100%和99.54%。市委、市政府12件27项为民办实事项目顺利完成。

各位代表，过去一年，我市经济建设和社会各项事业取得了显著进步，这是省委、省政府和市委正确领导的结果，是全市人民团结奋斗、开拓进取的结果。在此，我代表市人民政府向全市广大干部群众，各民主党派、工商联和无党派人士、各人民团体以及其他社会各界人士，驻泉省部属单位、人民解放军和武警部队，向为泉州建设作出贡献的企业界人士、外来务工人员，向所有关心、支持和参与泉州现代化建设的港澳台同胞、海外侨胞、海内外朋友致以崇高的敬意！对人大代表、政协委员和全市人民给予政府的信任和大力支持表示衷心的感谢！

在肯定成绩的同时，我们也清醒地看到，我市经济社会发展还存在一些困难和问题。主要是：农业增效农民增收仍较困难，农村社会事业投入不足，发展仍较滞后；产业结构调整和发展方式转变任务艰巨，第三产业发展相对滞后，现代服务业总量偏小；能源、原材料价格上涨和土地、资金、劳动力、人才、技术等要素瓶颈制约更加突出；中心城市辐射带动功能有待进一步增强；国际经济不确定因素增加，外经贸发展压力加大；环境保护、节能降耗和污染减排任务仍然较重；居民消费价格上涨幅度较大，城乡部分低收入群众生活水平受到影响；社会保障覆盖面还比较低，社会福利设施建设相对滞后；社会管理和公共服务水平有待提高，市区交通拥挤、农村群众就医和边远山区群众子女就学难等问题尚未根本解决；稳定低生育水平与统筹解决人口问题任务还十分繁重；安全生产形势仍较严峻；基层矛盾纠纷时有发生，社会治安防控体系仍需完善；个别公务人员行政不作为、乱作为现象依然存在，依法行政水平有待提高；反腐倡廉工作还需进一步加强。此外，2007年市《政府工作报告》提出的218项任务有4项尚未完成，15个年度预期指标有1项未达到预期要求，上述问题需要我们进一步采取措施，认真加以解决。

二、2008年政府工作的指导思想、预期目标和任务措施

2008年是我市深入实施“十一五”规划和全面建设小康社会的关键一年。综观国内外形势，世界经济有望保持增长态势；党的十七大胜利召开，中央进一步支持海峡西岸建设，泉州对台优势更加凸显，全市上下呈现团结拼搏谋发展的良好势头，泉州已经处在一个可以更好更快发展的关键时期。同时，我们也清醒地看到，受国际贸易摩擦加剧、汇率变动、价格波动、货币从紧、出口退税调整等因素影响，我市面临的国际国内竞争将更加激烈。我们必须把握机遇，立足实际，适应国内外形势的新变化，顺应全市人民过上更好生活的新期待，努力推动经济社会又好又快发展，在建设“科学发展的先行区、两岸人民交流合作的先行区”中更好地发挥排头兵作用。

2008年政府工作的指导思想是：全面贯彻党的十七大精神，高举中国特色社会主义伟大旗帜，以邓小平理论和“三个代表”重要思想为指导，深入贯彻落实科学发展观，按照省委八届三次全会、市委十届三次全会的统一部署，牢牢把握“四谋发展”实践主题、“四个重在”实践要领和“四个关键”工作要求，围绕“好字当头、先行先试”工作主线，加快转变经济发展方式，着力保障和改善民生，推进社会主义新农村、经济强市、现代城市、文化名城、和谐泉州、生态文明建设取得新成效，又好又快建设海峡西岸经济区现代化工贸港口城市。

2008年我市经济社会发展主要预期目标为：全市生产总值增长14.5%；财政总收入增长17%，其中一般预算收入增长17%；规模以上工业总产值增长22%；全社会固定资产投资增长23%；出口商品总值（海关口径）增长15%；实际利用外资（验资口径）增长13%；第三产业增加值增长15%；社会消费品零售额增长16.5%；居民消费价格总水平涨幅控制在4.5%左右；城镇居民人均可支配收入增长9%，农民人均纯收入增长7%；城镇登记失业率控制在2%以内；人口自然增长率控制在8.6‰以内；万元生产总值能耗下降3.5%，二氧化硫排放量削减4.2%，化学需氧量排放量削减1.1%。

实现上述目标和要求，我们要扎实抓好以下工作：

（一）以农民增收为核心，致力推进社会主义新农村建设

强化支农惠农政策落实。加大“三农”投入，今年市本级安排“三农”资金1.94亿元，增长15.6%，在建设安排、分配政策、财政支出、民生保障上向农村和老少边岛地区倾斜。认真执行粮食订单收购直接补贴和最低收购价政策，切实抓好种粮、良种、农机等补贴资金的落实，保护种粮农民积极性。发展农民专业合作社，推广农业产业化新型利益联合机制，加快农业社会化服务体系建设。加强对农村土地承包经营权流转的指导和监管，鼓励有条件的村和乡（镇）发展多种形式的适度规模经营。继续深化集体林权制度改革。认真落实农村信用社改革扶持政策。改进支持服务农村的方式，抓好省级乡（镇）“三农”服务中心试点，健全“六大员”制度。

大力发展现代农业。坚持把发展现代农业、繁荣农村经济作为“三农”工作的首要任务。加强标准农田建设和中低产田改造，牢守粮食种植面积280万亩底线，防止抛荒、撂荒。按照“抓龙头、建基地、打品牌、拓市场、深加工、扶行会”要求，深入实施“1333”工程，扶持19家省级、104家市级农业产业化龙头企业发展，培育无公害农产品生产基地20个、绿色食品生产基地10个、有机食品生产基地3个。依托全国农产品展览会、“茶博会”、“花博会”、“林博会”等平台，大力推介泉州知名农产品，推进“泉州龙眼”区域品牌建设，发展农产品商标及地理标志产品。做好高致病性禽流感、高致病性猪蓝耳病、柑桔黄龙病等重大动植物疫病和外来有害生物的防控工作。发展海洋捕捞、海水养殖和水产品加工等蓝色产业，加快建设深沪、祥芝、崇武国家中心渔港。继续推进碧坑水库项目前期工作。

提高新农村建设水平。总结推广“百企联百村”、“文化先导型新农村”等经验，抓好20个市级新农村建设试点示范村、20个市级农村社区试点村。办好新农村建设“十件实事”：提级改造建制村公路 100公里 ；建设村级供水工程200个；加固山围塘480座；建立完善农家店1800家；培训农村劳动力5万人，转移农村劳动力6万人；新增10个沼气建设示范村、10个生态家园示范点和10个沼气协会；深入开展“家园清洁行动”，完成26个乡（镇）、350个建制村的垃圾站建设；完成20户以上已通电自然村“村村通”广播电视工作；帮扶10个省级、20个市级扶贫开发工作重点村；实施“造福工程”，实现搬迁5000人目标。

（二）以产业升级为主轴，致力推进经济强市建设

全力打造先进制造业基地。加快推进新兴产业“十大载体”建设，认真做好产业链的规划和延伸，做大做强五大新兴产业。电子信息业重点要建设好江南高新技术电子信息产业园区、南安光电信息产业基地、洛秀园区、惠安绿谷光电产业基地、泉州软件园，认真落实扶持和促进光电信息技术产业发展的意见，着力抓好金太阳、三晶硅、金保利能源、长照科技、和谐光电等科技项目。石油化工业重点要建设好泉港和泉惠石化园区，力争炼化一体化炼油项目早日投产，继续实施中化重油深加工、石狮PTA等项目。汽车制造业重点要建设好泉州特种汽车基地，推进锐驰汽车电子、五洲龙汽车制造项目，鼓励一汽新华旭、远东北方奔驰汽车扩大生产。修船造船业重点要建设好惠安船舶配套基地，强化泉州船厂修造船项目的龙头拉动作用。生物医药业重点要建设好永春生物医药园区，切实抓好天馨生物科技等项目。同时，要加快福建省装备制造业（晋江）重点基地和太古复合材料项目建设，培育发展装备制造和新材料等产业。积极运用先进适用技术和信息技术，进一步整合提升五大传统优势产业，加强纺织、陶瓷2个省级行业技术开发基地建设，认真组织实施一批传统产业提升项目，下大力气开发新型高档面料、推广新型工艺技术，力争通过省级新产品鉴定20项。提升100家重点工业企业和60个省级新增长点企业的运行质量，努力培育一批规模上10亿元、20亿元、50亿元的大企业、大集团。认真实施“规模以下企业成长工程”，推动200家以上有潜力的规模以下企业通过技术进步、增资扩营，成长为规模以上企业。大力实施“回归工程”。鼓励优质企业在泉州设立总部、异地设立分部，把根留在泉州。

大力发展现代服务业。认真制订落实服务业主要行业年度发展目标，强化消费拉动，不断提高第三产业比重。改造提升商贸业，推进实施“万村千乡市场工程”，扩大农村消费市场，加快城东商业中心和泉州肉品加工厂建设步伐。整合提升交通运输等传统服务业。加快发展现代物流业，编制石湖、肖厝、斗尾港后物流区规划，抓紧推进石狮服装城（三期）、海峡西岸国际采购与区域物流中心、江南新区商贸物流中心、洛江仓储配送中心等物流项目建设。促进金融保险业发展，鼓励金融机构增加有效信贷投入，支持保险公司拓宽业务领域，为地方经济建设提供优质服务。积极培育新兴服务业，规范发展会展、会计、信息咨询、中介服务、业务外包、法律服务、社区服务等产业，加快发展工业设计、动漫、软件等创意产业。引导房地产业健康发展。进一步壮大旅游服务业，抓好南少林寺旅游片区、惠安崇武旅游度假区、泉美植物园等项目建设，加快星级酒店建设步伐，大力发展文化旅游和滨海风情旅游。

完善开放型经济体系。不断提高利用外资质量，加强外资项目库建设，着力引进有利于产业升级的骨干项目，力争出口加工区在招商选资上有较大突破。做好保税港和保税物流中心（B型）申报工作。鼓励企业扩大急需原材料、关键设备和关键技术进口。积极转变外贸增长方式，用好用活外贸发展专项资金，积极培育海关总署“红名单”企业，加快国有外贸企业改制步伐，扩大自主品牌产品出口，争取安溪国家级茶叶重点实验室和福建石油化工检测中心实验室通过核查验收，引导企业有为应对国际贸易摩擦和国际市场波动。办好“鞋博会”、“石博会”、“网洽会”等经贸活动。认真实施“走出去”战略，鼓励企业到境外设立营销网络，跟踪菲律宾晋江经济贸易区筹建情况。同时，要不断深化泉港、泉澳经贸合作；做好侨务工作，加强与华侨、华人及其新生代的沟通联系，关心贫困归侨、侨眷的生产生活，推进华侨农场的改革和发展。

提升泉台合作交流水平。推动泉台区域合作先行先试，全力打造台资聚集区。认真实施《泉台产业对接专项规划》，办好“产洽会”、“海博会”和台湾农产品采购订货会等对台经贸活动，力争在引进台湾电子信息、机械、石化、物流产业等方面取得新突破。积极跟踪台商投资区报批，建设好惠安黄塘台商创业基地等载体，加快在谈和签约大项目的落地投产。进一步扩大直接往来成果，争取泉金航线开展“包裹”业务，建设好福建闽台农产品交易市场，不断做大对台小额贸易。加强泉台农业合作，组织实施《海峡两岸（泉州）农业合作试验区发展规划》。深化泉台文化交流，推动泉澎两地旅游协议落实，打响郑成功文化品牌。

推进体制改革与自主创新。全面推进信息化建设和信息化管理机构改革，进一步完善全市信息化发展规划，科学整合、提升、建设信息化公共服务平台，大力发展电子政务、电子商务，加快国民经济和社会信息化步伐。完善国有资产监督管理体制，逐步推进城镇集体企业改革，抓好港口开发建设有限公司等组建工作。深化投融资体制改革，推行政府投资项目代建制，规范项目工程招投标行为，探索和建立网上招投标、网上采购制度，进一步规范公共资源市场化配置。健全服务机制，推动企业依法上市、规范上市，打造“泉州板块”。构建中小企业信用担保体系。加快自主创新步伐，完善科技创新奖励政策，实现市、县两级财政科技投入2.87亿元。启动新一轮科技重大专项工作，实施科技计划项目200项。强化企业创新主体地位，新增省级以上高新技术企业15家、重点技术创新项目30个。加强研发机构建设，新增市级以上工程技术研究中心和行业技术研究中心10家、省级以上企业技术中心8家。认真实施知识产权战略，推进版权工作，确保专利申请和授权量增长10%以上。大力实施品牌带动战略，争创更多的中国驰名商标、中国名牌产品、出口免验产品，力争参与制定修改国家、行业标准15项，采用国际标准和国外先进标准15项。密切产学研合作，充分发挥“6·18”项目成果交易会平台作用，完善区域自主创新体系。

（三）以重点项目为载体，致力推进现代城市建设

完善城市交通能源体系。推进基础设施建设“四大工程”，构建方便快捷的现代综合交通网络。高速铁路工程要加快福厦铁路泉州段建设步伐，做好向莆铁路湄洲湾南岸支线前期工作。高速公路工程要确保泉三高速公路泉州段主体完工，加快泉厦和福泉高速公路扩建、泉三高速公路南惠支线建设进度及沈海高速公路复线南安（金淘）至厦门（集美）段开工，抓紧做好环城高速公路（二期）晋江至石狮段、双永高速公路泉州段前期工作。桥梁通道工程要确保泉州晋江大桥通车，继续做好泉州湾跨海通道前期工作。电力电网工程要加快南埔电厂（二期）、晋江燃气电厂、石狮鸿山热电厂和泉州500千伏输变电工程、泉州城网改造工程建设步伐。

塑造现代城市崭新形象。深入推进“一二三四”城建项目建设。城东片区要围绕完善全国农运会主场馆及相关配套设施，确保滞洪排涝工程和滨江路等20条市政道路建成，加快福炼总部、美仙山安置房（一期）封顶和其他安置小区建设步伐。东海片区要加快综合大道、景观东西道、滨城大道连接线等四条市政道路及沿海大通道丰泽段（三期）建设步伐，争取宝秀、海星安置小区（一期）工程基本完成，开工建设东海滩涂整理项目。桥南片区要认真做好大部分安置房的交房回迁工作，实现已拍卖地块开工建设和南低渠、华洲渠改造竣工。北峰组团要尽快组织分区规划和控制性详规，抓紧泉州火车站综合交通枢纽开工，做好站前大桥前期工作。洛秀组团要抓紧完成百崎湖修建性详规，开工建设南北主干道。认真执行市十四届人大一次会议议案决议，深入开展中心市区道路交通综合整治。大力发展城市公交，加强公交站场规划建设，优化拓展公交线路，强化出租车行业管理。继续推进中心市区路网改造建设，确保田安路南拓、南俊巷北拓道路通车，加快洛阳江滨江大道建设步伐，着手进行城市轻轨交通规划的前期工作。完善市政设施，加快泉州市液化天然气配套管网和金浦水厂建设步伐，做好北峰供水加压站及配套管网建设前期工作。实施古城危旧房屋加固和翻建规划，坚决制止“两违”，加快市容市貌及景观整治，提高城市品位。各县（市）要主动对接泉州中心城市建设，认真执行《泉州市市域城镇体系规划》，合理布局，因地制宜，建设各具特色的县城及重点乡（镇），科学配置公共基础设施，做到互相衔接、资源共享、优势互补。

认真实施港城联动战略。争取《泉州市港口总体规划》获批，控制、保护、使用好岸线资源和港口后方陆域。大力推进港口基础设施建设，确保肖厝10万吨级多用途码头、石湖10万吨级集装箱码头、鲤鱼尾成品油码头建成或投产，加快泰山石化10万吨级码头及湄洲湾、泉州湾、围头湾航道工程建设，做好秀涂“人工岛”开发建设可行性研究及一批深水码头建设的前期工作，争取完成年度投资10.7亿元。推进湄洲湾船舶交管系统、泉州海巡基地、泉州溢油应急设备库建设，确保航运安全。认真落实港口生产优惠政策，加大港口推介力度，鼓励港航企业开辟新航线，积极吸引本外地货源从泉州港进出，大力推进海铁联运，力争港口货物吞吐量完成7300万吨、集装箱完成120万标箱。完善“大通关”机制，加快电子口岸建设步伐，提高港口服务水平。

（四）以传承创新为动力，致力推进文化名城建设

办好第六届全国农运会。全面完成泉州市海峡体育中心、泉州迎宾馆（一期）及比赛场馆、集训基地的建设、改造和维护，确保按时交付使用。精心做好开幕式、闭幕式文体表演筹备和赛事组织服务。积极选拔优秀运动员和教练员，力争完成省下达的夺金任务。深入开展“活力泉州迎农运，道德新风伴我行”主题活动，加强产业、文化和城市品牌推介，展示城市良好风貌。认真做好接待服务、医疗卫生、安全保障、市场开发、财务管理和媒体转播等各项配套工作。强化会务组织管理，努力把本届农运会办成专业水平高、服务质量佳、“海西”特色鲜明的农民体育盛会。

提升泉州文化软实力。切实把社会主义核心价值体系融入文化建设全过程，努力提高全市人民的思想道德文化素质，创建（2006—2008年度）省级文明城市。立足国家首批历史文化名城定位，继续推进“海上丝绸之路”申报世界文化遗产、“南音”申报“世界非物质文化遗产代表作”；出台实施《闽南文化生态（泉州）保护实验区规划纲要》和非物质文化遗产传承人保护规定；推进东西塔保护、承天寺景观修复工程建设，建成清净寺新礼拜堂，启动泉州木偶剧院建设，完成泉州歌剧团选址立项；认真做好第二轮修志工作，抓紧编撰《泉州文库》和《泉州南音集成》。坚持为人民服务方向，加强社区文化、企业文化和校园文化建设；深入开展文化“三下乡”和“万千百十”活动，推进实施农村电影“2131”工程，壮大村文化协管员和文化辅导员队伍；建好50个乡（镇）综合文化站、500个村文化活动室、50个文化信息资源共享点和150家“农家书屋”。实施艺术精品战略，创编、上演人民群众喜闻乐见的剧目，打造具有泉州特色和全国影响的文化艺术精品。深化文化体制改革，大力发展文化产业，促进泉州文化事业新发展。

加快教育强市和人力资源强市建设步伐。坚持教育公益性质，加大财政对教育的投入。坚持教育公平，促进义务教育均衡发展，全部免除城乡义务教育阶段学杂费，免费为农村义务教育阶段学生提供教科书，落实补助农村义务教育阶段寄宿生生活费政策，保障经济困难家庭和外来务工人员子女平等接受义务教育。推进农村中小学布局调整，扶持15所农村寄宿制小学配套建设。加强素质教育，新增洛江实现省“双高普九”。加快普及高中阶段教育，大力发展职业教育，加强中等职业学校实训基地和专业建设。支持办好辖区内各高等院校。重视学前教育，关心特殊教育、老年教育，加强社区教育、青少年校外教育，发展远程教育和继续教育，构建学习型社会。继续加强人才工作，认真落实人才政策，实施“桐江学者计划”，加 强企业 博士后科研工作站建设，推进高校毕业生“三支一扶”计划，继续做好赴外地开展人才招聘工作，积极引进高层次人才、留学人员和紧缺人才，为我市经济社会发展特别是新兴产业发展提供人才支撑。

（五）以改善民生为重点，致力推进和谐泉州建设

认真实施积极的就业政策。完善就业创业服务体系，抓好农村劳动力培训转移，完成培训各类劳动力25万人、新增就业15万人任务。继续加强跨区域劳务合作。坚持以创业带动就业，切实落实小额担保贷款等支持自主创业、自谋职业政策。及时帮助零就业家庭解决就业困难，认真做好大中专毕业生就业指导和服务工作。积极构建和谐劳动关系，认真实施《劳动合同法》，落实带薪休假政策，健全企业职工工资正常增长机制。

加快建设覆盖城乡居民的社会保障体系。健全“五险统征”机制，扩大城镇职工保险覆盖面。完善新型农村合作医疗制度，力争全市农民参合率达90%。实施“金保工程”。启动城镇居民基本医疗保险，力争参保人数达20万人以上。继续推进外来务工人员、农民工住院医疗保险，完善机关事业单位养老保险制度，出台实施被征地人员养老保险指导意见，探索建立农村养老保险制度。落实粮食储备任务，稳定市场物价，保障粮油副食品供应。大力发展慈善事业，加快构建覆盖城乡的新型社会救助体系。做好优抚对象、“五老”人员、“五保户”和残疾人、孤残儿童等特殊困难群体的福利保障工作。加快江南、见龙亭及各县（市、区）经济适用房和廉租房建设，今明两年建设1000套廉租房，逐步解决城市低收入家庭住房困难。

提高人民群众健康水平。完善公共卫生体系，增强重大疾病防控和突发公共卫生事件应急处置能力。加强农村卫生工作，推进乡（镇）卫生院体制改革、危房改造和应急救护能力建设，制定农村卫生组织吸引和留住人才的措施。加快市第一医院新院、福建医大附属二院新院和泉州医疗中心建设步伐，完成市人民医院整体搬迁，推进20个重点乡（镇）卫生院配套建设。进一步完善中心市区医疗急救网络，提升社区卫生机构服务水平，加强对民营医院、诊所的管理，加快建立覆盖城乡的基本医疗卫生制度。深入开展爱国卫生运动，迎接国家卫生城市复评。加强食品药品安全监管体系建设，在全市村（居）聘用食品药品质量安全协管员，巩固发展产品质量和食品安全整治成果。深化整顿规范市场秩序工作。进一步做好人口和计划生育工作，继续稳定低生育水平，综合治理出生人口性别比失衡问题，强化流动人口计生管理服务，提高出生人口素质。深入开展“生育关怀行动”，加强妇幼卫生保健工作，促进妇女儿童事业全面发展。

维护社会安定稳定。深化“平安泉州”建设，推进社会治安综合治理，严厉打击各种违法犯罪活动，有效防控多发性案件，提高群众安全感。继续完善“多证合一、多举并重、多位一体”的流动人口服务管理新机制。健全预防化解矛盾纠纷机制，更加注重人文关怀和心理疏导，妥善解决人民群众来信来访突出问题。坚持安全发展，强化企业安全生产主体责任，全面推进重点行业、重要领域安全专项整治和市县重大危险源监控系统建设，坚决遏制重特大安全事故发生。做好防震减灾和抗震设防工作。完善突发公共事件应急管理机制，不断提高应急管理和有效防范各种风险的能力。

切实加强双拥工作。统筹经济建设和国防建设，做好国防动员、民兵、预备役工作，认真落实和完善各项优抚政策，妥善安置退伍、转业军人和随军家属，继续争创全国双拥模范城，巩固军政、军民团结的大好局面。

（六）以节能减排为抓手，致力推进生态文明建设

大力倡导生态文明观念。全面落实市委、市政府《关于进一步加强环境保护工作的决定》，把建设资源节约型、环境友好型社会放在更加突出位置。各级政府要率先垂范，牢固树立生态文明观，建设节约型政府，推进生态市建设。企业要强化节能环保意识和主体责任，自觉推行清洁生产和ISO14000环境管理体系认证。全体市民要爱护生态、崇尚自然，坚持适度消费、绿色消费，使建设生态文明成为每一个泉州人的共同价值和自觉行动。

努力推进资源能源节约。认真实施十大重点节能工程，加强电力、煤炭、冶金、石化、建材、轻纺等重点耗能行业和企业的节能监督管理，抓好100家重点耗能企业节能工作，推进节能科技进步，坚决淘汰落后产能。认真实施20个循环经济示范项目，加快可再生能源开发，壮大循环经济规模。加强水资源配置管理与节约保护，统筹解决沿海地区用水紧张问题。实行最严格的耕地保护政策，强化用地监管，严肃查处违法违规用地行为。继续实施连片万亩土地整理项目，大力推进土地节约集约利用，盘活闲置土地，启动农村宅基地整理复垦试点工作，全面推行工业用地“招拍挂”。各县（市、区）要有意识预留一批工业用地，为高新技术产业发展留足空间。

切实加强生态环境保护。认真执行市十三届人大七次会议议案决议，进一步加强近海水域环境污染治理，认真实施81个年度计划整治项目。深化晋江、洛阳江流域环境污染综合整治，完善上下游生态补偿机制，保护和改善山美水库等水源水质。全面推进10吨以上燃煤锅炉和二氧化硫年排放量100吨以上企业的脱硫设施建设，基本完成火电、建陶企业除尘脱硫设施改造。完善市、县两级环境自动监测监控系统，抓好93家市级重点污染源监管。认真做好泉州市第一次全国污染源普查工作。基本建成城东、石狮（二期）、晋江仙石（二期）、惠南工业区污水处理厂，加强城市污水管网和中水回用设施建设，提高城市污水处理厂运营管理水平。全面开征垃圾处理费，推进县、乡（镇）、村垃圾收集转运处理三级管理体系建设，加快泉州、石狮（二期）、晋江（二期）、南安、惠安、安溪垃圾焚烧发电厂和永春垃圾填埋场建设步伐。力争中心市区生活污水处理率、垃圾无害化处理率和集中式饮用水水源水质达标。全面实施矿山生态环境恢复治理保证金制度，加强地质灾害的防治工作。开展“生态植树年”活动，广泛发动群众植树造林，推进生态公益林保护和水土保持工作，做好迎接国家园林城市复检工作。

三、有效作为，提高服务型政府建设水平

推进我市经济社会又好又快发展，任务十分繁重。各级政府要牢固树立“以人为本、执政为民”理念，不断加强自身建设，增强为人民服务的本领，科学运作，有效作为，努力建设一个人民群众放心、满意、信赖的政府。

坚持科学发展。牢牢把握发展第一要义，用科学发展观指导政府行为、检验工作成效。强化决策的科学性，坚持重大事项集体研究决定，重视听取方方面面意见，确保政府决策符合科学发展观要求。强化工作的协调性，把握好经济社会发展必须注意的“八个方面问题”，促进经济社会持续协调健康发展。强化措施的长远性，牢记社会主义初级阶段基本国情，多做一些利于子孙后代、利于可持续发展的事。强化制度的保障性，建立健全落实科学发展观的体制机制和考评体系。

强化公共服务。切实转变政府职能，健全公共服务体系，完善公共财政框架，增强提供公共卫生、义务教育、社会保障、环境保护等公共产品和服务的能力，促进基本公共服务均等化。重视解决涉及人民群众切身利益的民生问题，抓好市委、市政府11件27项为民办实事项目，认真解决群众反映的热点难点问题。继续完善领导接访群众制度，进一步建设好“政风行风直通车”、“一线通”、“政风行风热线”等平台，畅通民意表达渠道，体察人民所急、所需、所求，实现好、维护好、发展好人民群众的根本利益。

力求务实高效。加强政府管理创新，在政府工作的各个环节实行精细化管理。全面推行企业服务项目全程式网上审批，完善“绿色通道”，规范县、乡（镇）两级行政服务中心运作，健全三级审批联动机制，进一步促进审批提速。牢固树立服务意识和责任意识，加强部门工作的协同与合作。建立行政问责制，切实解决行政不作为、乱作为问题，确保政令畅通。深化机关效能和绩效评估工作，建立办事高效、运转协调、行为规范的行政管理体系。

推进依法行政。弘扬法治精神，全面落实依法行政实施纲要，扎实开展“五五”普法，加快依法治市进程。深入贯彻《监督法》，认真执行人大及其常委会决议和决定，自觉接受人大及其常委会的法律监督和工作监督，认真办理人大代表建议。重视和支持人民政协履行政治协商、民主监督、参政议政职能，认真办理政协委员提案。接受新闻舆论监督和社会公众监督。认真实施《政府信息公开条例》，拓展政务公开渠道，推进诚信政府建设，提高政府工作透明度和公信力。认真推行行政执法责任制，加强行政执法能力建设。进一步推进规范行政处罚自由裁量权和相对集中行政处罚权工作。

保持清正廉洁。坚持把反腐倡廉建设放在更加突出位置，完善教育、制度、监督并重的惩治和预防腐败体系。深入贯彻中纪委《关于严格禁止利用职务上的便利谋取不正当利益的若干规定》，严格执行党风廉政建设责任制，坚决查处违法违纪案件。积极推进“公共服务伦理规范与廉政建设”项目试点工作，进一步纠正部门和行业不正之风。认真执行《关于严格控制行政支出建设节约型机关的若干意见》，从严控制接待费、会议费、公车费和公费出国出境，规范机关公务用车，反对铺张浪费，勤俭办一切事业，今年公用经费定额实现零增长。加强行政监察、财政监督、审计监督和政府督查工作，加强对工程建设招投标、经营性土地使用权出让、国有资产运营、政府采购、行政审批等领域的监管，深入开展商业贿赂专项整治工作。

各位代表，党的十七大提出了夺取全面建设小康社会新胜利的宏伟目标，为我们踏上新的历史征程指明了方向，泉州进入了又好又快发展的新阶段。让我们更加紧密地团结在以胡锦涛同志为总书记的党中央周围，高举中国特色社会主义伟大旗帜，在市委领导下，全面贯彻落实科学发展观，为推进海峡西岸经济区现代化工贸港口城市建设而努力奋斗！
